# FACSNet: Forensics aided content selection network for heterogeneous image steganalysis

**DOI:** 10.1038/s41598-024-77552-x

**Published:** 2024-11-01

**Authors:** Siyuan Huang, Minqing Zhang, Yongjun Kong, Yan Ke, Fuqiang Di

**Affiliations:** 1 College of Cryptographic Engineering, Engineering University of PAP, Xi’an, 710086 China; 2 Counterterrorism Command & Information Engineering Joint Lab in Urumqi Campus, Engineering University of PAP, Urumqi, 830000 China

**Keywords:** Steganography, Steganalysis, Deep learning, Forensics aided, Content selection, Computer science, Information technology

## Abstract

The main goal of image steganalysis, as a technique of confrontation with steganography, is to determine the presence or absence of secret information in conjunction with the specific statistical characteristics of the carrier. With the development of deep learning technology in recent years, the performance of steganography has been gradually enhanced. Especially for the complex reality environment, the image content is mixed and heterogeneous, which brings great challenges to the practical application of image steganalysis technology. In order to solve this problem, we design a forensics aided content selection network (FACSNet) for heterogeneous image steganalysis. Considering the heterogeneous situation of real images, a forensics aided module is introduced to pre-categorise the images to be tested, so that the network is able to detect different categories of images in a more targeted way. The complexity of the images is also further analysed and classified using the content selection module to train a more adapted steganalyser. By doing this, the network is allowed to achieve better performance in recognising and classifying the heterogeneous images for detection. Experimental results show that our designed FACSNet is able to achieve excellent detection performance in heterogeneous environments, improving the detection accuracy by up to 7.14% points, with certain robustness and practicality.

## Introduction

With the rapid development of the internet, information transfer and communication has become very convenient, and a large amount of digital information is widely distributed and used. Rapid advances in digital communications technology and huge increases in computer power have led to an exponential growth in the use of the Internet and in the transmission of all kinds of complex data and multimedia objects. Securing the content of sensitive and personal transactions on open networks is therefore critical but increasingly challenging. To protect information privacy, researchers have studied and developed communication security mechanisms. Steganography provides two of the most obvious solutions. Encrypting secret information Converts it into observable but meaningless noise-like data, thereby masking the existence of the secret message^1^. Unfortunately, due to the emergence of image steganography, lawbreakers can maliciously transmit information through images, causing ad verse effects on society and national security. The principle is to modify the data of an image with the help of the insensitivity of optic nerve perception function, and hide secret information into the image without affecting the original visual effect. Since image steganography attempts to make secret information look like random image noise generated by camera sensors and circuitry, detecting such malicious tampering with the human eye alone is impossible. Therefore, researchers have developed image steganalysis techniques for detecting and combating this threat.

Image steganalysis and image steganography are technologies that act against each other and promote development of each other in gaming^2,3^. Steganalysis, the opposite of steganography, has been proposed to detect whether an image is embedded with secret information. The difficulty of image steganalysis is that the steganalysis model cannot fully and effectively model the slight differences that occur when the steganography operation embeds secret messages in an image. Image steganalysis has experienced a development process from traditional to deep learning. The rich model proposed by Fridrich et al.^4^ is a typical representative of traditional steganalysis, which combines various functions and uses an integrated classifier for training. In the JPEG domain, some modern schemes, such as DCTR (Data Content Type Recognition)^5^ and SCA-GFR (Selection-Channel-Aware Gabor Filter Residual)^6^, extract features from the residuals of the decompressed JPEG images, achieving good results. However, these models also face many difficulties. First, the algorithm design is complicated. Second, adjusting feature parameters requires considerable amount of time and energy, leading to low experimental efficiency.

In recent years, the development of deep learning has unified and automated the two steps of feature extraction and classification in traditional image steganalysis, enabling end-to-end methods and achieving satisfactory results. Qian et al.^7^ proposed a network structure with five convolutional layers, using the KV kernel as the preprocessing layer to preprocess the images, allowing the model to directly learn the residual images and reduce the interference of the image content in training. XuNet, proposed by Xu et al.^8^, used the KV kernel as a high-pass filter layer for image preprocessing. The network used five convolutional layers, and the convolution kernel size of the first two layers was 5$\times$5. Subsequently, Xu et al.^9^ proposed a model specifically for detecting JPEG steganography, referred to as J-XuNet herein. This architecture relies on the fixed preprocessing of the DCT (Discrete Cosine Transform) kernels in the first convolutional layer and thresholding its feature maps. Ye et al.^10^ proposed YeNet, which uses a deeper ten-layer convolutional network structure and 30 SRM (Spatial Rich Model) convolution kernels as preprocessing layers to allow the model to learn more features. Chen et al.^11^ proposed PNet\Vnet, which modified XuNet for the steganalysis of JPEG images by splitting the feature maps into 64 parallel channels to make the architecture aware of the JPEG phase the underlying grid of 8$\times$8 pixels. Fridrich et al.^12^ proposed the SRNet which comprises four convolutional layer modules with different functions, effectively using the BN (batch normalization) layer^13^ and residual network. Further, it adds channel selection to improve the detection accuracy of the steganography algorithms. Notably, this model provides state-of-the-art detection accuracy for both the spatial domain and JPEG steganography. Zeng et al.^14^ proposed WISERNet for steganalysis of color images, which preserves strong correlation patterns while damaging uncorrelated noise, effectively reducing the complexity of model detection performance gains. Zhu et al.^15^ proposed a new CNN network structure that uses separable convolution to utilize channel correlation of residuals to compress image content and improve signal-to-noise ratio, while using data enhancement technology to improve detection accuracy of spatial domain steganalysis. Liu et al.^16^ proposed a steganalysis network CSANet which introduces a residual channel spatial attention (CSA) module to acquire knowledge for selective channel perception. Spatial pyramid pooling was also used to further improve the performance of JPEG steganalysis. Fu et al.^17^ considered that existing steganalysis models lack attention to regional features with complex textures, which affects the detection accuracy of steganalysis. They designed an image steganalysis model based on channel attention mechanism, and guided the model to focus on useful features.

Generic steganalysis and specialized steganalysis are the two main approaches in the field of steganalysis^18^. Generic steganalysis does not depend on specific steganographic algorithms, so it can detect a variety of steganographic algorithms and has strong generalization ability. Since it does not target specific steganographic algorithms, generic steganalysis can be useful in a variety of application scenarios and is suitable for practical application needs. With the development of technology, the detection accuracy of generic steganalysis has gradually approached that of specialized steganalysis, becoming an important research direction in the field of steganalysis. However, since generic steganalysis needs to learn statistical models from a large number of samples, there may be model mismatch problems for unknown data, resulting in a decrease in detection accuracy. Meanwhile, in order to ensure the generality, generalized steganalysis usually needs to deal with more data and features, so the computational complexity is relatively high. Dedicated steganalysis analyzes and tests known steganographic algorithms, and because of the a priori knowledge of steganographic algorithms, the detection accuracy is usually higher. At the same time, it can be optimized for specific steganographic algorithms to improve the relevance and effectiveness of detection. When new steganographic algorithms appear, specialized steganalysis may not be able to immediately adapt and effectively detect them, and requires updating the algorithm or retraining the model. We believe that a combination of generalized steganalysis and dedicated steganalysis can be adopted as a solution. In practical applications, the training model can choose the appropriate steganalysis method according to the specific needs and scenarios, so that it has a strong generalization ability.

Most of the existing research on image steganalysis uses a single image library (e.g. Bossbase 1.01, BOWS2) in the experiments, mainly because of its grayscale and consistent-size image format that facilitates experimental observation and comparison. However, in the actual network environment, the source, quality, content, and processing of images are complex and diverse, existing steganalysis models rarely consider the content of the images, so the steganalysis model is often difficult to achieve ideal detection results due to the mismatch problem in the face of such mixed heterogeneous images, which leads to a significant reduction in the effectiveness of the existing image steganalysis systems in practical applications. It is worth mentioning that, in the real world, cover images may have been processed intentionally or unintentionally, so some steganalysis models will judge processed cover images as stego images, resulting in a high false alarm rate of cover images in steganalysis. In recent years, many studies have shown that forged images whose contents have been tampered with can be misjudged as stego images by steganalysis models, which greatly reduces the accuracy of steganalysis algorithms^19^. Therefore, it is of great theoretical significance and application value to carry out research on steganalysis techniques for mixed heterogeneous image sources.

In this paper, we propose a forensics aided content selection network (FACSNet) to address the problem of performance misalignment of steganalysis in heterogeneous environments. We observe that if steganalysis detection is performed only in a single laboratory environment, the model is prone to mismatch problems due to overfitting, which in turn prevents efficient processing and detection of complex images in real-world environments. Therefore, we designed heterogeneous datasets to simulate the complex environment in reality. At the same time, the forensics aided module and the content selection module are added to the backbone network to guide the network to adopt more targeted schemes for different classes of input images, thus increasing the robustness and practicality of the model. Note that the environments we simulate all make use of publicly available datasets and algorithms. We have designed FACSNet to be tested in a realistic web environment with a targeted approach, and we believe that it is a worthwhile endeavor to advance image steganalysis techniques from a laboratory setting to a real-world environment. And extensive experiments on these datasets show that our proposed FACSNet has excellent detection performance in heterogeneous environments.

The rest of the paper is organised as follows. The “Methods” section describes the basic principles of our proposed FACSNet and its algorithmic details. The “Results and Discussion” section conducts detailed experiments to validate our ideas and the performance of FACSNet, and discusses and analyses them. Finally, the “Conclusion” section summarises our research and gives an outlook for future work.

## Methods

### Forensics aided module

The development of deep learning has brought huge performance improvements in image steganalysis models. The process of model construction requires three main phases: training, validation, and testing. Indeed, in most cases, the training process of the steganalysis model is crucial, where the features of the input training set represent the statistics of the images that the steganalysis model mainly deals with. This issue is often overlooked or underestimated in current research, yet a mismatch between the statistics of the training and test sets can significantly degrade the performance of a steganalyzer. It is worth noting that nowadays complex network environments with mixed heterogeneous image features exist. If we only use the same kind of training set to train the model, then the model may not work well in the testing phase, and more likely to encounter mismatches in real applications.

**Figure 1:**
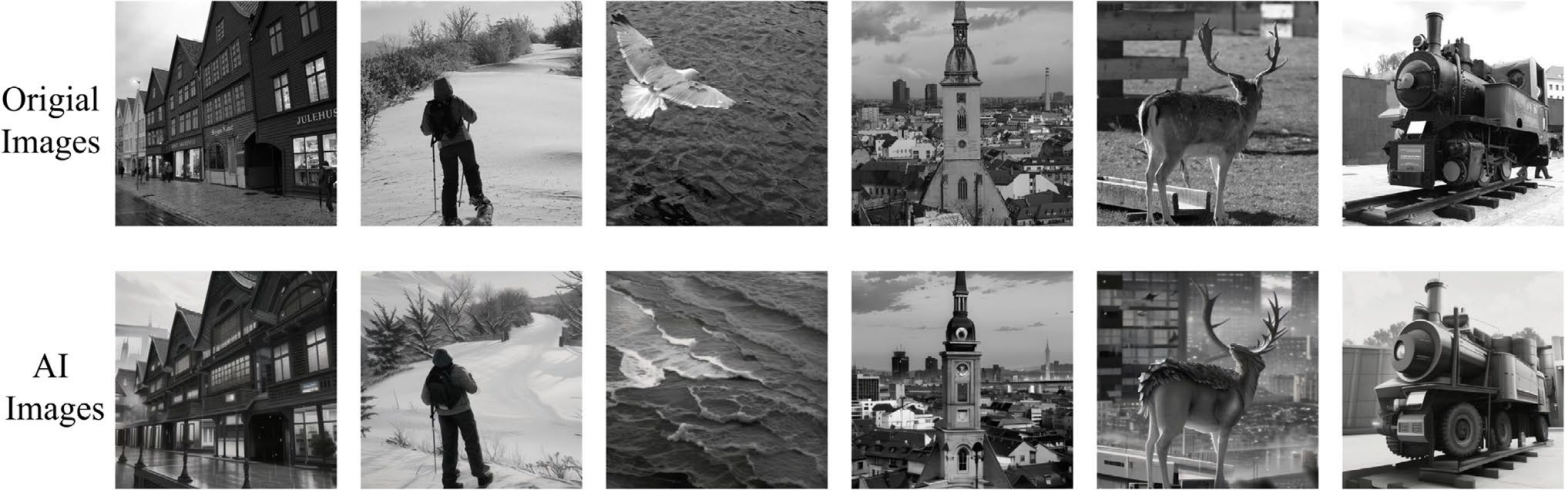
Examples of the two types of images. The first row is the original image from the Bossbase dataset and the second row is the corresponding AI-generated image.

In this paper, we have designed a preclassification module that maximizes the performance of the steganalysis model when images are preclassified as different classes in the mixed heterogeneity at the time of input. The basic idea of the module is that there is a classification step at the time of image input in which the images are categorized into different types. The different types of images are then analyzed separately using multiple versions of the steganalysis models trained on different types of image training sets. At the same time, we consider constructing a hybrid heterogeneous data environment to simulate a real-life situation. In order to validate our algorithm in a relatively simple situation, we simulate real images into two categories, the original image acquired by the camera and the computer-generated image (AI image). We simulate the above two types of images with original images from the Bossbase dataset and AI-processed original images. For AI generation of images we used the Dreambooth^20^ model released by Google that currently works well. Examples of the two types of images are shown in Fig. 1. These two image types were chosen mainly because the statistical differences between the two types of images are significant, thus allowing for the presence of more significant realistic representations in the steganalysis of mixed heterogeneous images.

**Figure 2:**
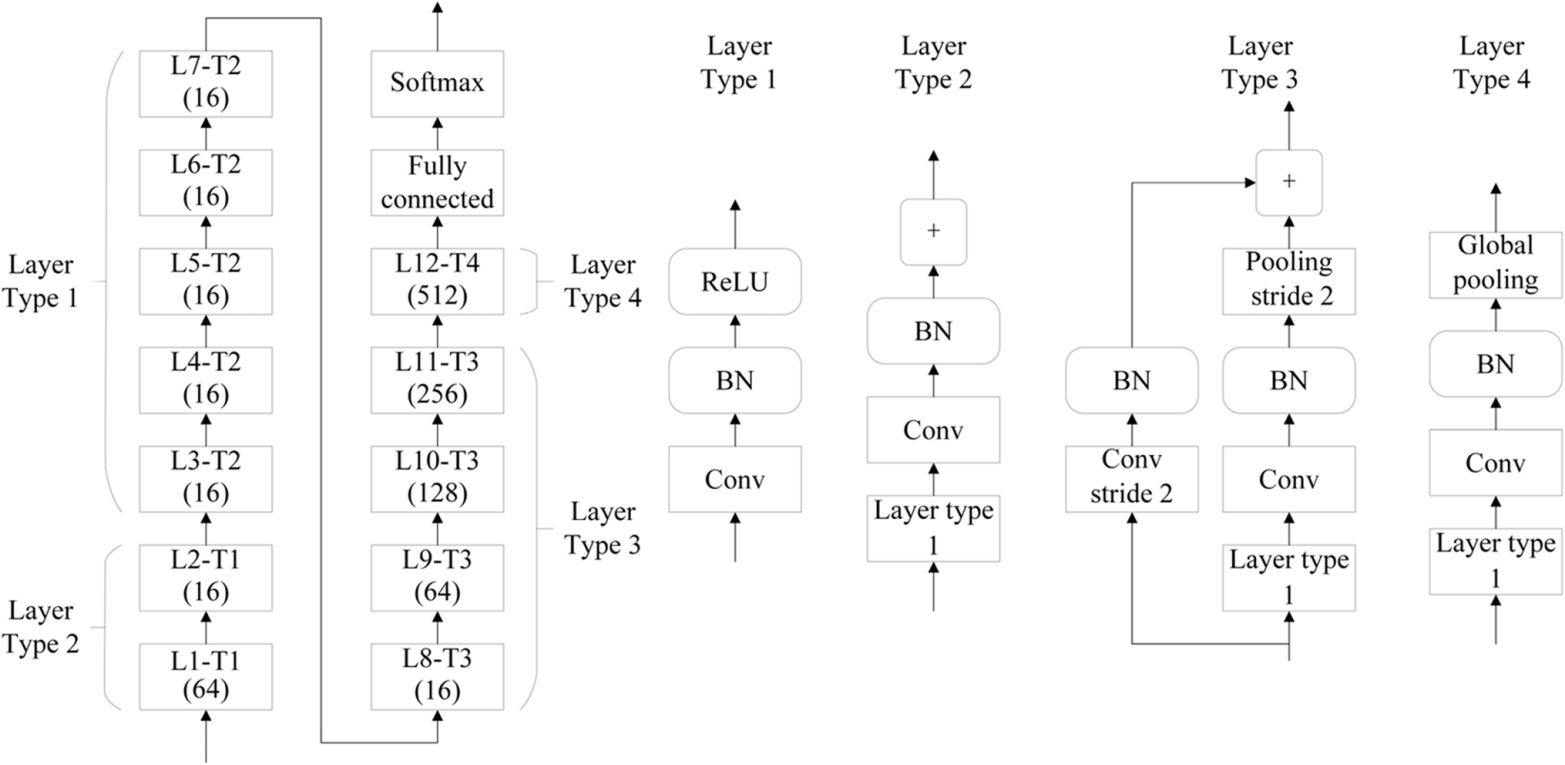
Structural diagram of the network model used in the forensics aided module.

Based on the above idea, we construct a of steganalysis Forensics Aided module that utilizes the properties of the image forensic task to discriminate mixed heterogeneous image sources. We can also call it a transforming steganalysis module because it can transform between different kinds of corresponding steganalyzers depending on the class of the image to be analyzed. In the training phase of the model, we designed datasets 1 and 2 containing both types of original and AI images, and then use the steganalyzers to train the image sets of different classes, and named the corresponding steganalyzer as *S*_*1*_, *S*_*2*_. Here we use the backbone network of the classical SRNet model to train the original image and the AI image, so that the trained model can distinguish the two types of images well, and the structure of the network model is shown in Fig. 2. Model is mainly composed of four parts. The first part (Layer Type 1) is to replace the traditional pre-processing stage with convolution. The second part (Layer Type 2) is responsible for extracting noise residual in the image. The third part (Layer Type 3) is mainly to reduce the dimension of the feature map, and the last part (Layer Type 4) mainly uses a standard full connection layer and a softmax node to classify the results.

It is worth noting here that we have chosen only two types of images in order to validate our algorithm at the lowest energy consumption. In practical applications, *N* classes can be selected. In the testing phase of the model, the input mixed heterogeneous images are firstly used to discriminate which possible class they belong to by using forensic model, and then the version of the corresponding trained steganalyzer is used.

### Content selection module

When testing the forensic aided module, we found that the category recognition of the image alone is not enough to fully capture the steganographic signals in a targeted manner, so we designed a content selection module to further analyze the image content information. Existing image steganalysis is gradually starting to pay attention to the content of the image, Amrutha et al.^21^ proposed a novel MixNet framework consisting of six convolutional neural networks as a feature extractor for accomplishing generalized steganalysis of spatial content adaptive algorithms with higher detection accuracy, Arivazhagan et al.^22^ proposed a hybrid deep learning framework to model the noise residuals and extract five hand-crafted features dedicated to the design of the network structure; Amrutha et al.^23^ proposed a novel deep clustering network DCNSD based on a convolutional autoencoder and clustering model, which is able to distinguish images transmitted by a steganographer from those of an innocent user in an unsupervised manner. However, we found that the types of images in reality are far more complex than the set of images usually applied in experiments. Especially for the image adaptive steganography algorithms, which are now more difficult to detect, it is possible to reduce the difficulty of steganalysis if more consideration is given to the behavior of the steganographer in the premask selection process.

Feature extraction of an image is an important basis for content selection, in which texture features of an image are important for describing the content of the image. The texture feature of an image describes the surface properties of the image scene and is a value calculated from the image, which contains a large amount of useful information and reflects the spatial distribution properties of the pixels. In existing adaptive image steganography algorithms, the secret information is usually embedded in complex texture regions to enhance its masking, because the distortion caused in complex texture regions is less likely to be detected by the detector.

Textures can be categorized into three types in real life: natural textures, artificial textures and mixed textures. Natural texture is an attribute formed naturally by objects in nature, usually with a large variety of elements, no regularity, irregular shape, such as leaf texture, tree texture, cloud and smoke texture. Artificial texture is an attribute of objects formed through artificial participation, usually constituting the texture of the elements are more regular, with a certain degree of regularity, such as fabric patterns and wall texture. Mixed texture mainly refers to the artificial participation in the manufacture, mixed texture is mainly an attribute of objects without regularity, characterized by a disorderly arrangement of the constituent elements, such as artistic graffiti and artistic texture. In order to provide an intuitive basis for the training of the model, the images in our study are generally categorized into two classes based on the content complexity: complex images and simple images. Considering that the textures of the cover images vary, we apply the Gray-Level Co-occurrence Matrix (GLCM)^24^ to the content selection module as a way to measure the texture complexity of the image and analyze it. GLCM can reflect comprehensive information such as the direction, adjacent intervals, and range of variation of the image gray scale.

**Figure 3:**
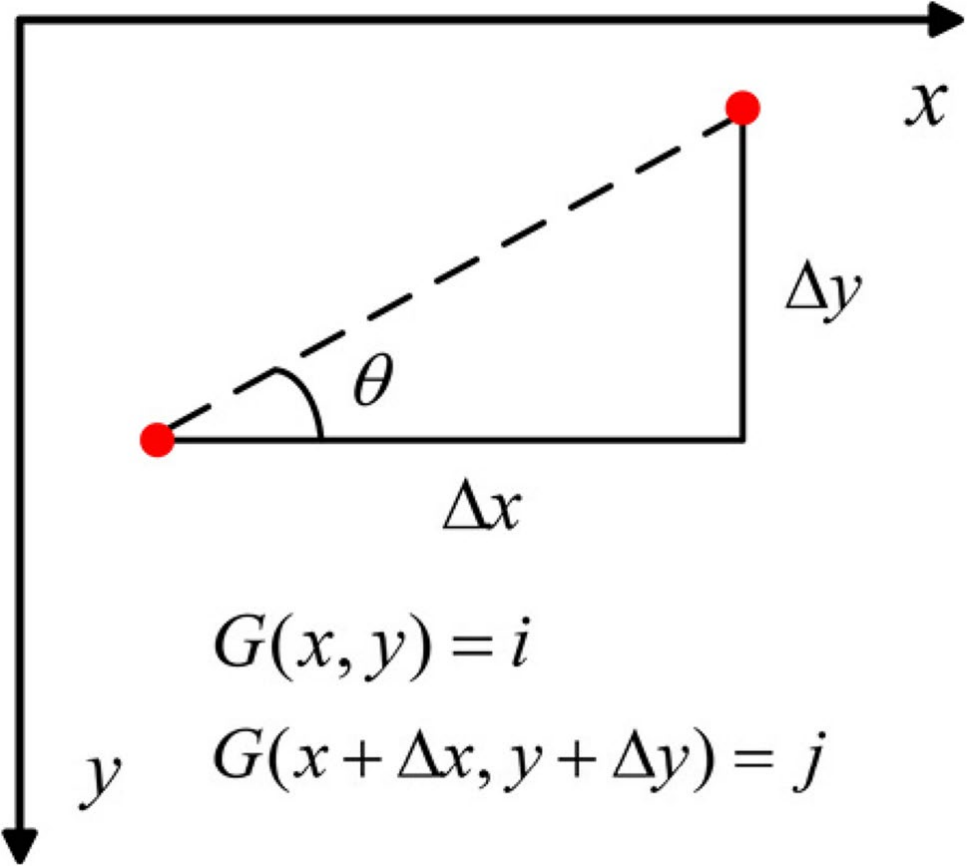
Relationship between pixel pairs.

The GLCM index and the eigenvalues based on GLCM can quantify the image texture complexity. The joint probability distribution of the simultaneous occurrence of two pixel pairs with gray values *i* and *j* is called the gray level covariance matrix, which can be expressed as $P(i,j)(i,j=1,2,3\dots,N)$. Where $P(i,j)$ denotes the probability of a pixel to go from gray level *i* to gray level *j* in the original image, where *N* denotes the gray level of the image. The correspondence between *i* and *j* satisfies $D=(\Delta x,\Delta y)$, which determines the distance and direction information between two pixels. Then the joint probability distribution can be represented by a gray level covariance matrix of order $N \times N$as shown in Fig. 3.

In Fig. 3, $\theta$ denotes the angle of the GLCM scanning direction. $G(x,y)$ denotes the grayscale value of the pixel at the position $(x,y)$ and $G(x+\Delta x,y+\Delta y)$ denotes the grayscale value of the pixel at the position $(x+\Delta x,y+\Delta y)$. Traversing all pixels in an image *N* will produce $N \times N{\text{ }}[G(x,y),G(x+\Delta x,y+\Delta y)]$ binary combinations. Arrange all occurrences of $[G(x,y),G(x+\Delta x,y+\Delta y)]$ pairs into a matrix. Assuming that $t{\text{ }}[G(x,y),G(x+\Delta x,y+\Delta y)]$ is the number of occurrences of the value pair, the total number of occurrences *T* in all cases is denoted by:

1 $$T=\sum\limits_{{i=1}}^{N} {\sum\limits_{{j=1}}^{N} {t[G(x,y),G(x+\Delta x,y+\Delta y)]} }$$

The elements in the matrix ${P_{N*N}}$ are denoted by:

2 $$P{\text{ [}}G(x,y),G(x+\Delta x,y+\Delta y)]=\frac{{t[G(x,y),G(x+\Delta x,y+\Delta y)]}}{T}$$

3 $${P_{N*N}}=\left[ \begin{gathered} P(1,1){\text{ }}P(1,2){\text{ }} \cdot \cdot \cdot {\text{ }}P(1,N) \hfill \\ P(2,1){\text{ }}P(2,2){\text{ }} \cdot \cdot \cdot {\text{ }}P(2,N) \hfill \\ {\text{ }} \cdot \cdot \cdot {\text{ }} \cdot \cdot \cdot {\text{ }} \cdot \cdot \cdot {\text{ }} \cdot \cdot \cdot \hfill \\ P(N,1){\text{ }}P(N,2){\text{ }} \cdot \cdot \cdot {\text{ }}P(N,N) \hfill \\ \end{gathered} \right]$$

The GLCM can be obtained. Based on the GLCM, we compute a series of features related to it to reflect the spatial correlation of gray levels and improve the ability to describe texture. Here, we briefly define the four most common features:

1. Contrast (Con): Contrast is used to describe the local variations present in an image, the larger the contrast, the deeper the texture furrows and the clearer the visual effect. Conversely, the smaller the contrast, the shallower the furrows and the blurrier the effect. The larger the value of the elements in the GLCM that are far away from the diagonal, the larger the contrast is. So the larger the contrast the clearer the image. The calculating formula is as follows:

4 $$Con=\sum\limits_{i} {\sum\limits_{j} {{{(i - j)}^2}P(i,j)} }$$

(2) Homogeneity (H): Homogeneity reflects the magnitude of localized changes in the image texture; the inverse variance will be larger if it is more homogeneous and slower to change. Contrary to contrast or dissimilarity, the weight of homogeneity decreases with the distance of the element value from the diagonal in an exponential form. The calculating formula is as follows:

5 $$H=\sum\limits_{i}^{{}} {\sum\limits_{j}^{{}} {\frac{{p(i,j|D,\theta )}}{{1+{{(i - j)}^2}}}} }$$

(3) Angular Second Moment (ASM): Also called energy, it is a measure of the uniformity of image gray scale distribution. When the distribution of elements in the GLCM is more concentrated near the main diagonal, it means that the image gray scale distribution is more uniform in the local area, and the ASM takes a larger value accordingly; on the contrary, if all the values of the co-production matrix are equal, the ASM value is smaller. The calculating formula is as follows:

6 $$ASM=\sum\limits_{i} {\sum\limits_{j} {P{{(i,j)}^2}} }$$

(4) Entropy (Ent): Entropy is a measure of the randomness of the amount of information contained in an image. Entropy is maximum when all values in the covariance matrix are equal or when pixel values exhibit maximum randomness. Thus the entropy value indicates the complexity of the gray scale distribution of the image, the larger the entropy value, the more complex the image. The calculating formula is as follows:

7 $$Ent= - \sum\limits_{i} {\sum\limits_{j} {P(i,j)\log P(i,j)} }$$

**Figure 4:**
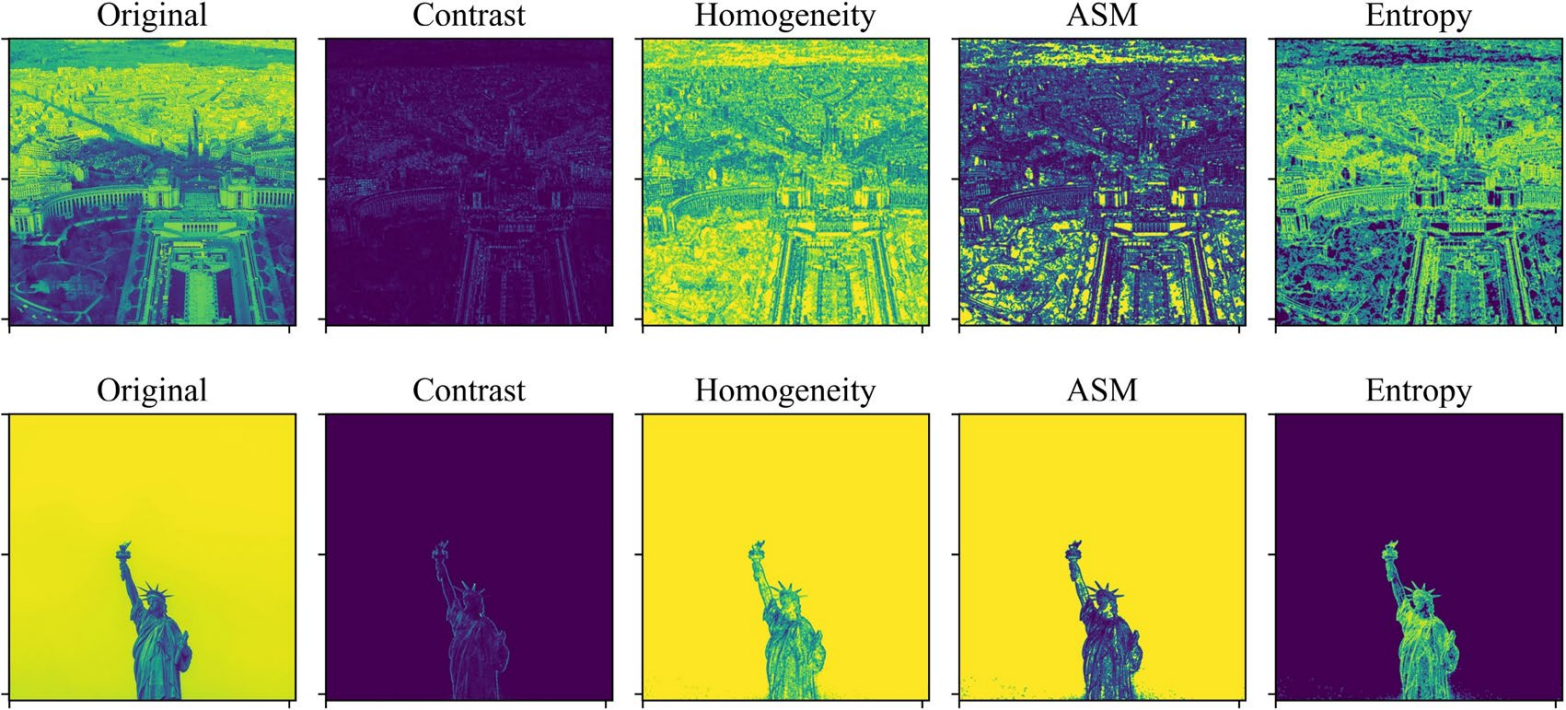
Visualization results for the four feature parameters of the GLCM. From the figure we can see that complex and simple images can exhibit large differences in feature parameters.

We visualize the four image attributes derived based on GLCM calculations as shown in Fig. 4. In order to better demonstrate the differences in attribute values between different images, we purposely selected two images with large texture differences for comparison. From the computational results, it can be seen that the attribute values of images with different texture complexity also have huge differences. Therefore, content selection is necessary for processing mixed heterogeneous images with different texture complexity. Ultimately, we define the normalized mean of the four feature vectors as the complexity *C* of the image and use this to classify the image. The calculating formula is as follows:

8 $$C=\frac{{\sum\limits_{n}^{{}} {[(x - \hbox{min} )/(\hbox{max} - \hbox{min} )]} }}{N}$$

Where *x* denotes the value of the relevant feature and N denotes the number of relevant features. Ultimately we sort the values and categorize the images into two categories of complex and simple images using bifurcation. The content selection module is implemented along the same lines as the forensic assistance module. We first divide the image as a whole into complex and simple images according to the image complexity, denoted by *A* and *B*, respectively. Secondly, we train the corresponding versions of steganalyzers *S*_*A*_ and *S*_*B*_ for the two types of images.

### FACSNet

**Figure 5:**
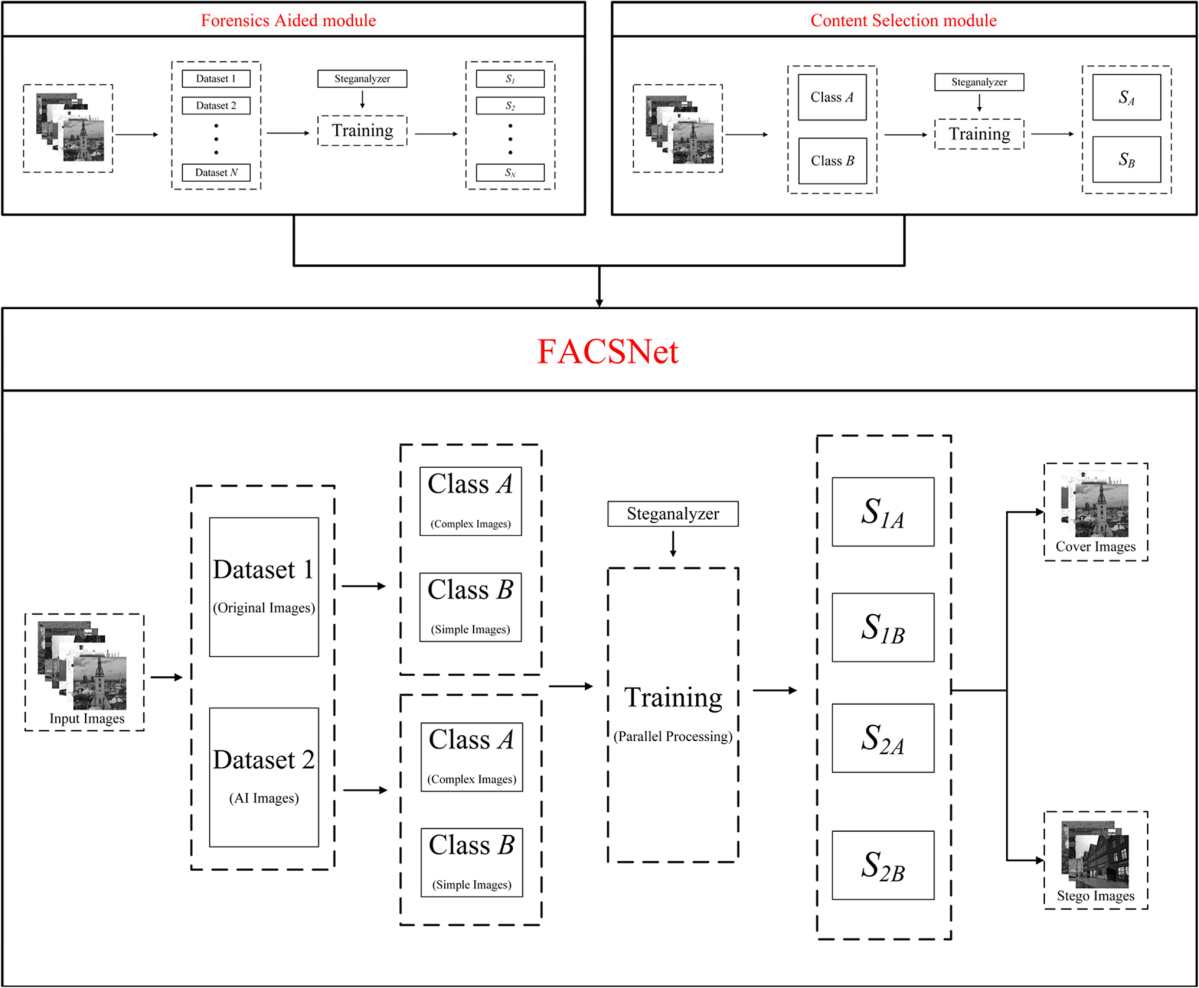
Overall scheme of FACSNet. The first half contains design ideas for two important modules of FACSNet. The second half is the general architecture of the combined FACSNet program.

In order to better capture the steganographic signals and maximize the effectiveness of the steganalysis model, we integrate the forensic-assisted module and the content selection module mentioned earlier, and design a forensics aided content selection image steganalysis network, which we call FACSNet. The network architecture of FACSNet is shown in Fig. 5, with the design ideas of the forensics aided module and the content selection module in the upper part, and FACSNet combining the two ideas in the lower part.

In FACSNet, firstly, the image is input into the forensics aided module to discriminate the kind of image, in our proposed algorithm, the input image can be categorized into original images and AI images, which corresponds to dataset 1 and 2. Secondly, the two types of images are then input into the content selection module respectively, and a second categorization is carried out based on the complexity of the image according to the selection algorithm that we have designed. This step of classification classifies the images into complex and simple images, which correspond to categories *A* and *B*. Up to this point, the image set as a whole is classified into four categories, i.e., *1 A*, *1B*, *2 A*, and *2B*. Finally, these four categories of the image set are trained in parallel using steganalyzers, which can result in four steganalyzer versions, *S*_*1A*_, *S*_*1B*_, *S*_*2A*_, and *S*_*2B*_. For prediction of unknown images, we input the image into FACSNet and firstly determine the kind of unknown image through forensic aided module. Secondly, the content complexity of the image is determined by the content selection module. Based on the above operations, the image can be categorized into one of the four types we defined earlier, e.g., *1 A*, and then we can use the *S*_*1A*_ steganalyzer to determine it. By using different versions of the steganalyzer for more targeted discrimination of different categories of images, we can efficiently and accurately perform steganalysis on mixed heterogeneous images and finally output classification results. In our scheme, the modeling algorithm mainly uses the general and efficient SRNet model. In practical and more complex environments, some existing forensic models can be used or borrowed, such as the artificial intelligence image detection algorithm proposed by Adobe^25^. It is important to point out here that the forensic algorithms and cryptanalysis models, while affecting the overall performance of FACSNet, are not the focus of our attention. Of course, we will update and improve the models therein in our future work to further improve their performance and usability.

## Results and discussion

### Experiments settings

To assess the robustness of the detector, the datasets BOSSbase 1.01^26^ and BOWS2^27^ were used for the performance evaluation, each containing 10,000 grayscale spatial images with a size of 512$\times$512. Their distributions were close. All images were resized to 256$\times$256 pixels using the MATLAB function imresize. The corresponding JPEG images were generated with quality factors (QFs) of 75 and 95. Four representative steganographic schemes, namely, HILL^28^ and HUGO^29^ for the spatial domain and UERD^30^ and J-UNIWARD^31^ for the JPEG domain, were attacking targets in the experiments. For spatial-domain steganographic algorithms, the embedding payloads were set to 0.1 to 0.4 bits per pixel (bpp). For JPEG steganographic algorithms, the embedding payloads were set to 0.1 to 0.4 bits per non-zero AC DCT coefficient (bpnzAC). In all the experiments, we initialized all channel scaling factors$\gamma$ = 0.5,$\alpha$= 10 − 4 to observe the benefits of FACSNet. All tests were performed using Tesla V100 and GeForce RTX3090 GPU cards.

In our experiments, we processed 5,000 images out of 10,000 images in the dataset as AI images, targeted them separately during training, and mixed them and then disrupted them during testing as a way to simulate the real-life mixed heterogeneous situation. Since the dataset images themselves have varying texture complexity, we do not preprocess the dataset too much in this section. The stochastic gradient descent optimizer Adamax was used with mini-batches of 16 cover-stego pairs. The training database was shuffled after each epoch. In our dataset, the training was run for 400k iterations with an initial learning rate of 0.001; subsequently, the learning rate was decreased to 0.0001 for an additional 100k iterations. During the comparison experiment, the method and comparison method proposed in this paper used the same training set, validation set, and test set, and the images of the three datasets were not repeated. The performance was evaluated by testing the accuracy and steganalysis error rates as follows:

9 $${P_{Accuracy}}=1 - {P_E}$$

10 $${P_E}=\frac{1}{2}\left( {{P_{FA}}+{P_{FN}}} \right)$$

Where ${P_{FA}}$ and ${P_{FN}}$ denote the probabilities of false positives and false negatives, respectively. The probability of the results was expressed as a percentage.

### Problem validation for steganalysis under heterogeneous environment

In this section, we first illustrate the importance and feasibility of our work by describing and validating the problem we are addressing in this paper. The main problem addressed in this paper is that the performance of image steganalysis in the mixed heterogeneous case can be degraded due to the mismatch problem. So we first train and test the steganalysis with the original BOSSbase dataset, and then test the steganalysis with the new dataset we constructed (we call it Heterogeneous-BOSSbase). We experimentally analyze them with two representative steganographic algorithms, HUGO and JUNIWARD, in the spatial domain and JPEG domain, respectively. The settings of the dataset and the experimental results of the steganalysis are shown in Table 1. From the results, we can see that if trained with a single dataset, BOSSbase 1.01, the model may perform well in the tests on the original dataset, but under the tests on the new heterogeneous dataset, it exposes its mismatch problem and the detection performance drops significantly. This is an important issue that we have been emphasizing that most of the existing techniques for image steganalysis have remained at the laboratory stage. So the work we carry out in this paper is relevant and can have some impact on the practicality of image steganalysis.

**Table 1:** Comparing the impact of implicit write analysis performance in the null and frequency domains for original and heterogeneous datasets.

Dataset	Content	Accuracy (%)	
		Spatial domain (HUGO-0.4bpp)	JPEG domain (JUNIWARD-0.4bpnzAC)
Bossbase 1.01	10,000 original images	90.64	91.75
Heterogeneous-BOSSbase	5,000 original images 5,000 AI images	85.76	88.30

### Training performance

**Figure 6:**
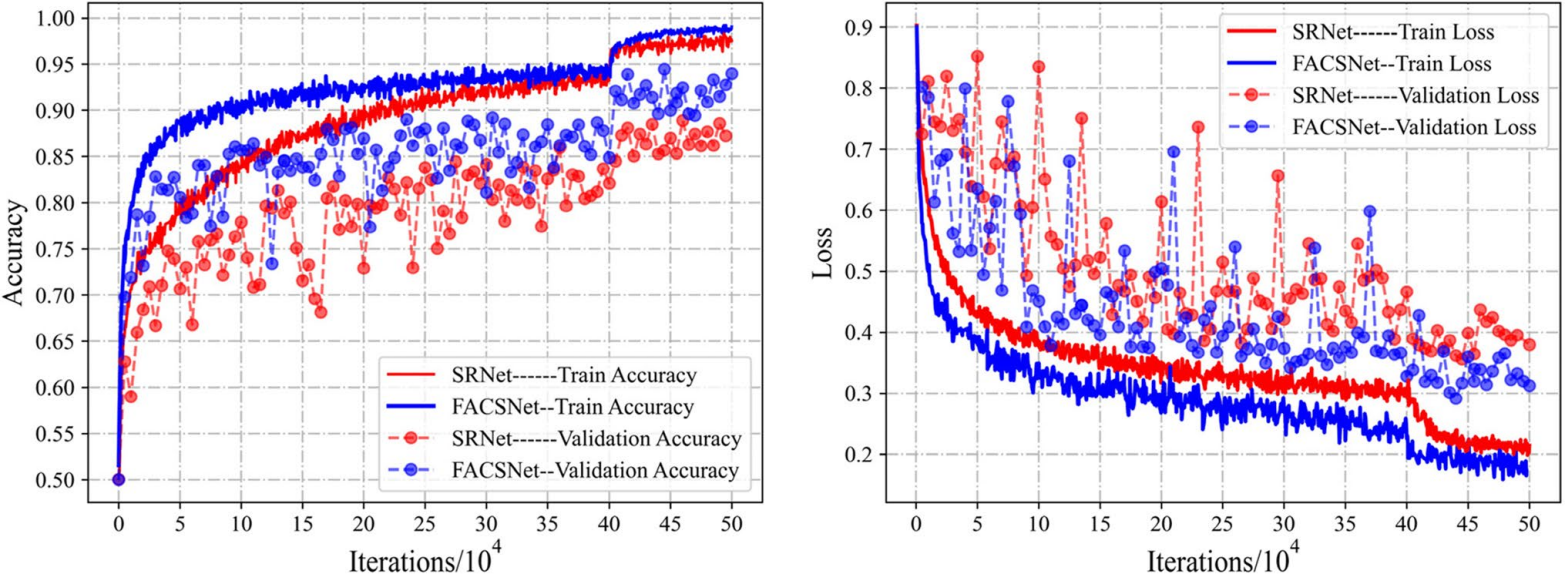
Detection accuracy and loss of FACSNet compared to SRNet in training.

For heterogeneous network image environments, we propose to use a parallel training approach to train a steganalyzer for each different image type that best applies to it, and jointly process it during the detection process to form a packaged overall architecture. The forensics aided module distinguishes the source category of the image and the content selection module distinguishes the content complexity of the image. The combination of the two modules ideally encompasses almost all realistic image categories. However, based on the complexity of the experiments, we first used the most convenient setup for validation. Meanwhile, we mainly reflect the overall gain of the model with the same backbone network in our experiments to highlight the feasibility of our scheme. Figure 6 shows the detection accuracy of our proposed FACSNet and a single SRNet for both the training and validation sets as well as the model loss during the training process. The steganography algorithm shown here for detection is JUNIWARD, with an embedding rate of 0.4bpnzAC and QF 75. From the accuracy plot, we can see that the performance of FACSNet improves very fast in the early stage of training, and can be stabilized at a level better than SRNet in the later stage. the loss plot also shows that FACSNet converges faster, which also verifies our previous idea. As the model is trained more diversely for its specialization, it essentially improves its generalization and usefulness. It should be noted here that FACSNet, due to its parallel structure, requires a more expensive time cost in the same experimental setting during training. However, the fact that FACSNet exhibits better detection performance and the detection speed remains consistent with the previous one makes us believe that these costs are worthwhile. Of course, we will further investigate and improve its training efficiency in our subsequent work.

### Detection performance

**Figure 7:**
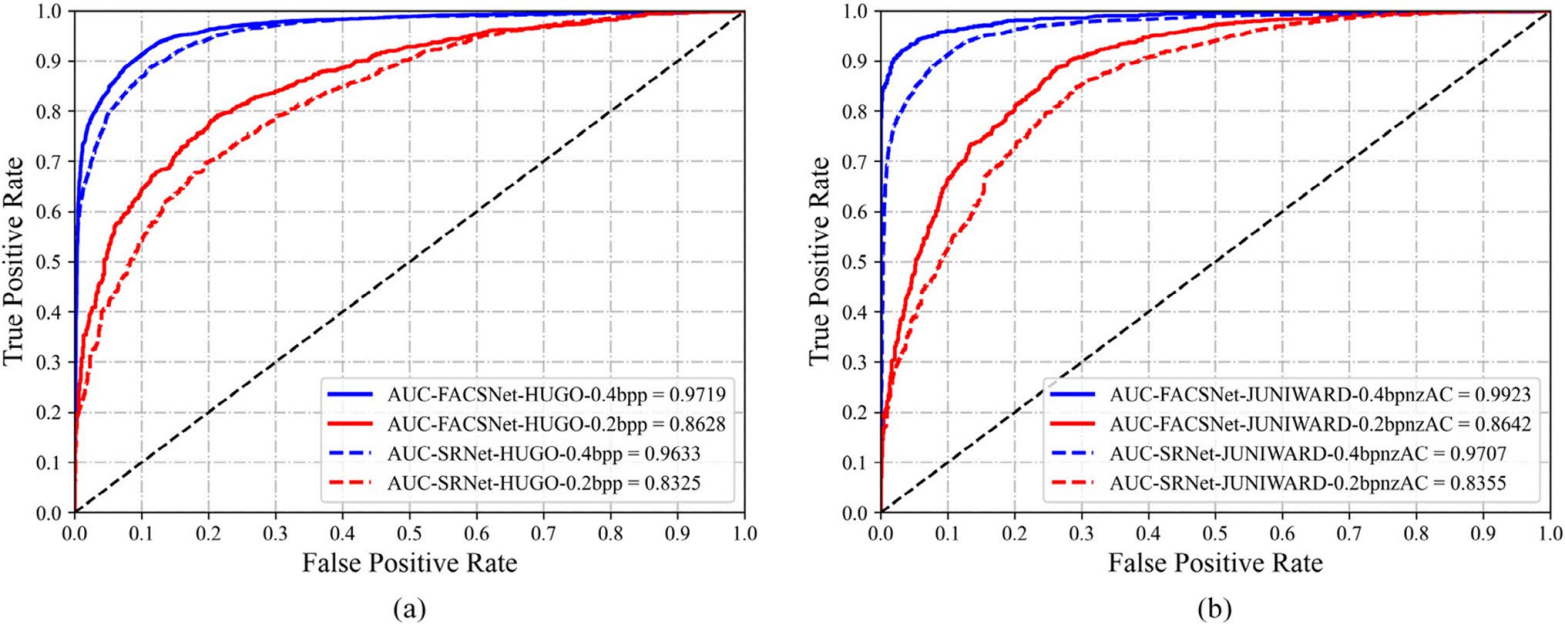
Comparison of testing accuracy of FACSNet and the corresponding original SRNet. (**a**) The trained models are aiming at detecting HUGO spatial-domain stego images with 0.2/0.4 bpp payload. (**b**) The trained models are aiming at detecting J-UNIWARD stego images with 0.2/0.4bpnzAC payload and QF 95.

To further validate the balanced performance of FACSNet in terms of sensitivity and specificity, we plotted the receiver operating characteristic (ROC) curve as shown in Figure 7. The closer the area under the ROC curve (AUC) value in the ROC curve is to 1, the better the model performance is represented. Overall, FACSNet exhibits competitive performance at different embedding rates.

**Table 2:** Comparison of detection accuracy between FACSNet and SRNet (JPEG domain) %.

Dataset	Steganography algorithm	Detection model	QF75				QF95			
			0.1 bpnzAC	0.2 bpnzAC	0.3 bpnzAC	0.4 bpnzAC	0.1 bpnzAC	0.2 bpnzAC	0.3 bpnzAC	0.4 bpnzAC
BOSSbase (All Original)	J-UNIWARD	SRNet	64.49	79.28	86.67	91.75	59.66	68.83	75.44	80.46
		FACSNet	64.49	79.31	86.67	91.75	59.66	68.83	75.44	80.46
	UERD	SRNet	81.61	89.85	94.33	96.58	67.60	78.41	85.73	89.38
		FACSNet	81.61	89.85	94.34	96.58	67.63	78.41	85.73	89.38
Heterogeneous-BOSSbase (Original + AI)	J-UNIWARD	SRNet	60.22	75.34	81.21	85.30	51.73	62.91	70.04	77.60
		FACSNet	64.38	78.85	86.63	91.73	58.35	68.56	75.09	80.37
	UERD	SRNet	75.83	86.66	89.01	93.18	61.25	72.91	81.46	86.27
		FACSNet	81.58	89.70	93.66	96.50	67.55	78.33	85.71	89.38
BOWS (All Original)	J-UNIWARD	SRNet	64.13	77.08	85.77	90.34	56.84	67.69	74.28	79.88
		FACSNet	64.13	77.09	85.77	90.34	56.85	67.69	74.32	79.88
	UERD	SRNet	80.77	89.48	93.60	95.43	67.55	78.03	85.34	88.69
		FACSNet	80.79	89.48	93.60	95.43	67.55	78.03	85.34	88.69
Heterogeneous-BOWS (Original + AI)	J-UNIWARD	SRNet	59.81	72.55	80.97	85.63	50.11	61.75	70.38	73.66
		FACSNet	63.39	76.80	85.52	90.18	56.34	67.65	74.19	79.83
	UERD	SRNet	74.07	84.81	88.62	91.83	62.33	70.80	80.77	85.04
		FACSNet	80.66	89.20	93.44	95.41	67.39	77.92	85.18	88.65

**Table 3:** Comparison of detection accuracy between FACSNet and SRNet (Spatial domain) %

Dataset	Steganography algorithm	Detection model	0.1 bpp	0.2 bpp	0.3 bpp	0.4 bpp
BOSSbase (All Original)	HILL	SRNet	65.53	73.41	79.72	84.25
		FACSNet	65.53	73.41	79.72	84.25
	HUGO	SRNet	71.44	80.52	86.33	90.64
		FACSNet	71.48	80.52	86.33	90.64
Heterogeneous-BOSSbase (Original + AI)	HILL	SRNet	60.04	69.80	75.11	80.63
		FACSNet	65.48	73.41	79.68	84.21
	HUGO	SRNet	66.96	75.56	82.07	85.76
		FACSNet	71.40	80.46	86.29	90.60
BOWS (All Original)	HILL	SRNet	62.87	71.82	78.44	82.23
		FACSNet	62.87	71.82	78.44	82.25
	HUGO	SRNet	70.53	79.66	85.01	89.88
		FACSNet	70.54	79.66	85.01	89.88
Heterogeneous-BOWS (Original + AI)	HILL	SRNet	57.45	66.47	73.93	78.06
		FACSNet	62.77	71.73	78.40	82.22
	HUGO	SRNet	64.60	74.85	80.55	83.52
		FACSNet	69.87	79.35	84.73	89.22

In Tables 2 and 3, we perform detailed experiments under different hyperparameters in the spatial domain and JPEG domain, respectively. For the dataset BOWS, we also take the same treatment as BOSSbase and name the new data as Heterogeneous-BOWS. By analysing the experimental results, we note that when the dataset is a single BOSSbase dataset, only the content selection module comes into play in FACSNet, which is then comparable to the performance of SRNet. However, there is a small performance improvement under some parameters due to its discriminative discrimination of images with different texture complexity. Notably, when the dataset becomes mixed and heterogeneous, FACSNet shows strong robustness and adaptability, and maintains good performance even when the performance of SRNet is significantly degraded. This is mainly attributed to the joint effect of our designed forensic assistance module and content selection module.

### Comparison with the state-of-art steganalyser

**Figure 8:**
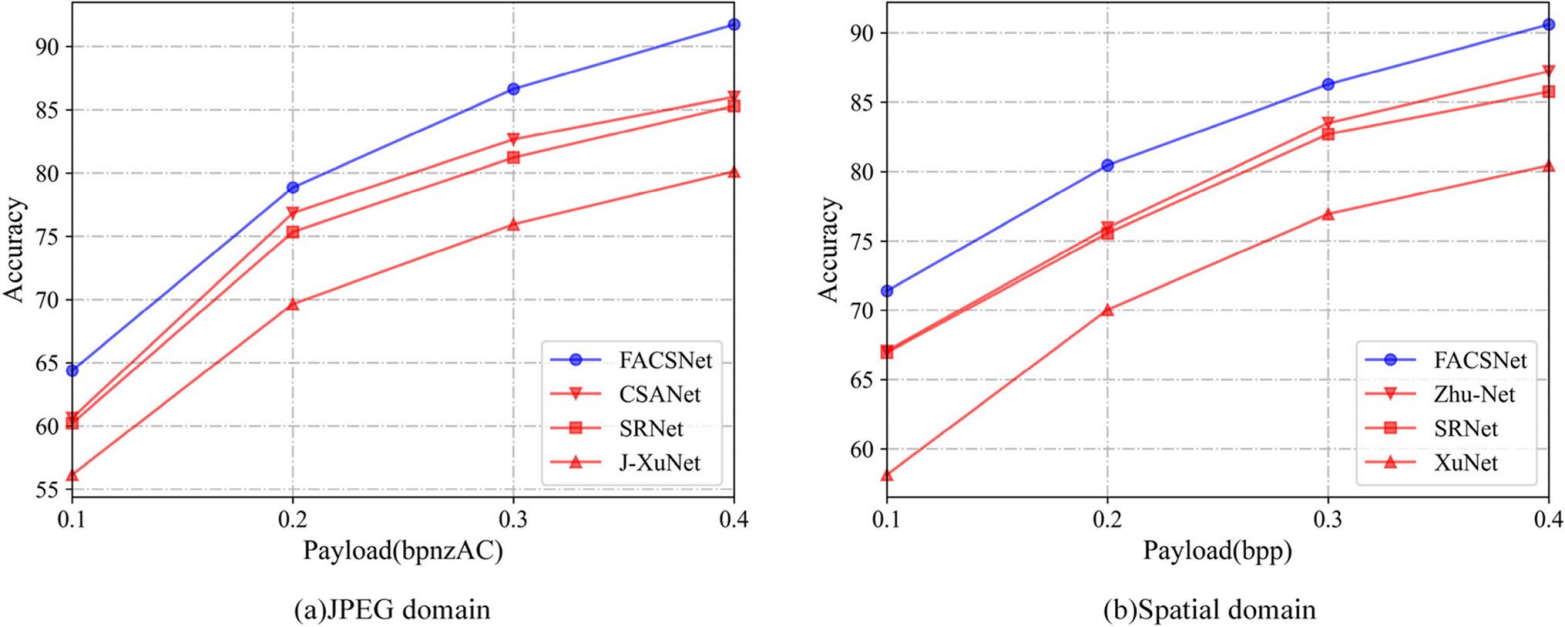
Comparison of testing accuracy of our proposed FACSNet with state-of-the-art steganalyzers in the literature. (**a**) Comparative situation in the JPEG domain. (**b**) Comparative situation in the Spatial domain.

To further validate the superiority of FACSNet performance, we compare the steganalysis performance of several state-of-the-art steganalysers in a heterogeneous environment. Among them, in the JPEG domain, we compared CSANet, SRNet and J-XuNet. in the air domain, we compared Zhu-Net, SRNet and XuNet. The results are shown in Fig. 8, which shows that FACSNet is clearly superior to other models in a heterogeneous environment. It should be noted here that FACSNet uses SRNet as the backbone network in this paper, so its base performance comes from the backbone network. Although effective gains are achieved in heterogeneous environments, it increases some of the losses in training time, and further attempts will be made to reduce the volume of the model and improve the efficiency of the model in our subsequent work.

## Conclusions

In this paper, we explore a new steganalysis architecture for solving the steganalysis mismatch problem in heterogeneous environments. We question the practicality of steganalysis, which is now restricted to the laboratory context, as it uses a single and more regular dataset, which is not compatible with the complex heterogeneous image environments in reality. We therefore simulate a heterogeneous dataset environment and train targeted steganalysis models for different input classes of images. The result is a steganalysis network that is capable of detecting multi-class heterogeneous images while maintaining the performance of the backbone network itself. Experiments show that our scheme can effectively improve the performance of the model for steganalysis in heterogeneous environments by up to 7.14% points in detection accuracy. However, we find that while the performance of FACSNet is improved, the training efficiency is reduced due to its parallel structure. In future research, we will further improve the training efficiency and detection performance of the network.

## Data Availability

The datasets used in the current study are publicly available. For BOSSBase 1.01, refer to https://dde.binghamton.edu/download/. For BOWS2, refer to https://drive.google.com/drive/folders/1VWE0X8TwpvTrdeTV2EPwyIeuXw80JxCc?usp=sharing/.

## References

[1] Abdulla, A. A. Exploiting similarities between secret and cover images for improved embedding efficiency and security in digital steganography. *The University of Buckingham*. (2015). http://bear.buckingham.ac.uk/149/. Accessed 12 Dec 2019.

[2] Priya, S., Abirami, S. P., Arunkumar, B. & Mishachandar, B. Super-resolution deep neural network (SRDNN) based multi-image steganography for highly secured lossless image transmission. *Sci. Rep.* **14**, 1. 10.1038/S41598-024-54839-7 (2024). 38480860 10.1038/s41598-024-54839-7 PMC10937672

[3] Eungi, H., KyungTae, L., Woo, T. O. & Haneol, J. Lightweight image steganalysis with block-wise pruning. *Sci. Rep.* **13**, 16148–16148. 10.1038/S41598-023-43386-2 (2023). 37752169 10.1038/s41598-023-43386-2 PMC10522667

[4] Fridrich, J. & Kodovsk´y, J. Rich models for steganalysis of digital images. *IEEE Trans. Inf. Forensics Secur.* **7**, 868–882. 10.1109/TIFS.2012.2190402 (2012).

[5] Holub, V. & Fridrich, J. Low-complexity features for JPEG steganalysis using undecimated DCT. *IEEE Trans. Inf. Forensics Secur.* **10**, 219–228. 10.1109/TIFS.2014.2364918 (2014).

[6] Denemark, T., Sedighi, V., Holub, V., Cogranne, R. & Fridrich, J. Selection-channel-aware rich model for steganalysis of digital images. In *6th IEEE International Workshop on Information Forensic and Security (WIFS’* 48–53. (2014). 10.1109/WIFS.2014.7084302 (2014).

[7] Qian, Y., Dong, J., Wang, W. & Tan, T. Deep learning for steganalysis via convolutional neural networks. In *IS&T/SPIE Electronic Imaging 2015 (Media Watermarking, Security, and Forensics)*. (2015). 10.1117/12.2083479

[8] Xu, G., Wu, H. & Shi, Y. Structural design of convolutional neural networks for steganalysis. *IEEE Signal. Process. Lett.* **23**, 708–712. 10.1109/LSP.2016.2548421 (2016).

[9] Xu, G. Deep convolutional neural network to detect J-UNIWARD. In *5th ACM Information Hiding and Multimedia Security Workshop (IH&MMSec’* 67–73. (2017). 10.1145/3082031.3083236 (2017).

[10] Ye, J., Ni, J. & Yi, Y. Deep learning hierarchical representations for image steganalysis. *IEEE Trans. Inf. Forensics Secur.* **12**, 2545–2557. 10.1109/TIFS.2017.2710946 (2017).

[11] Chen, M., Sedighi, V., Boroumand, M. & Fridrich, J. JPEG-phase-aware convolutional neural network for steganalysis of JPEG images. In *5th ACM Information Hiding and Multimedia Security Workshop (IH&MMSec’* 75–84. (2017). 10.1145/3082031.3083248 (2017).

[12] Boroumand, M., Chen, M. & Fridrich, J. Deep residual network for steganalysis of digital images. *IEEE Trans. Inf. Forensics Secu*. **14**, 1181–1193. 10.1109/TIFS.2018.2871749 (2018).

[13] Ioffe, S. & Szegedy, C. Batch normalization: Accelerating deep network training by reducing internal covariate shift. In *32nd Int. Conf. Mach. Learn. (ICML)*. 37, 448–456. (2015). 10.48550/arXiv.1502.03167

[14] Zeng, J. *et al*. Wider separate-then-reunion network for steganalysis of color images. *IEEE Trans. Inf. Forensics Secur.* **14**, 2735–2748. 10.1109/TIFS.2019.2904413 (2019).

[15] Zhang, R., Zhu, F., Liu, J. & Liu, G. Depth-wise separable convolutions and multi-level pooling for an efficient spatial CNN-based steganalysis. *IEEE Trans. Inf. Forensic Secur.* **15**, 1138–1150. 10.1109/TIFS.2019.2936913 (2019).

[16] Liu, Q., Ni, J. & Jian, M. Effective JPEG steganalysis using non-linear pre-processing and residual channel-spatial attention. In *2022 IEEE International Conference on Multimedia and Expo (ICME)*. 1–6. (2022). 10.1109/ICME52920.2022.9859742

[17] Fu, T. *et al*. CNN model with channel attention and convolutional pooling mechanism for spatial image steganalysis. *J. Vis. Commun. Image Represent*. 88. 10.1016/J.JVCIR.2022 (2022).

[18] Abdulla, A. A. Digital image steganography: challenges, investigation, and recommendation for the future direction. *Soft Comput.* **28**, 8963–8976. 10.1007/s00500-023-09130-8 (2024).

[19] Hou, X., Zhang, T., Xiong, G. & Wan, B. Forensics aided steganalysis of heterogeneous bitmap images with different compression history. *Ksii Trans. Internet Inform. Syst.* **6**, 874–877. 10.3837/tiis.2012.08.003 (2012).

[20] Ruiz, N., Li, Y., Jampani, V., Pritch, Y. & Dreambooth Fine tuning text-to-image diffusion models for subject-driven generation. (2022). 10.48550/arXiv.2208.12242

[21] Amrutha, E., Arivazhagan, S., Sylvia, L., Jebarani, W. & MixNet: A robust mixture of convolutional neural networks as feature extractors to detect stego images created by Content-Adaptive Steganography. *Neural Process. Lett.* **54**, 853–870. 10.1007/s11063-021-10661-0 (2022).

[22] Arivazhagan, S. *et al*. Hybrid convolutional neural network architecture driven by residual features for steganalysis of spatial steganographic algorithms. *Neural Comput. Applic*. **33**, 11465–11485. 10.1007/s00521-021-05837-7 (2021).

[23] Amrutha, E., Arivazhagan, S. & Jebarani, W. S. L. Deep Clustering Network for Steganographer Detection using latent features extracted from a Novel Convolutional Autoencoder. *Neural Process. Lett.* **55**, 2953–2964. 10.1007/s11063-022-10992-6 (2023).

[24] Haralick, R., Shanmugam, K. & Dinstein, I. Textural features for image classification. *Stud. Media Commun.*10.1109/TSMC.1973.4309314 (1973).

[25] Amini, S., Zhang, L. & Hao, B. An AI-assisted online tool for cognitive impairment detection using images from the clock drawing test. *Cold Spring Harbor Lab. Press.*10.1101/2021.03.06.21253047 (2021).

[26] Bas, P., Filler, T. & Pevn´y, T. Break our steganographic system-the ins and outs of organizing BOSS. In *13th Information Hiding Workshop (IH’2011)*. 59–70. (2011). 10.1007/978-3-642-24178-9_5

[27] Bas, P. & Furon, T. BOWS–2. (2019). http://bows2.ec-lille.fr

[28] Li, B., Wang, M., Huang, J. & Li, X. A new cost function for spatial image steganography. *In IEEE 2014 International Conference on Image Processing (ICIP’* 4206–4210. (2014). 10.1109/ICIP.2014.7025854 (2014).

[29] Pevný, T., Filler, T. & Bas, P. Using high-dimensional image models to perform highly undetectable steganography. In *12th Int. Workshop Inf. Hiding (IH)*. 161–177. (2010). 10.1007/978-3-642-16435-4_13

[30] Guo, L., Ni, J., Su, W., Tang, C. & Shi, Y. Using statistical image model for JPEG steganography: uniform embedding revisited. *IEEE Trans. Inf. Forensics Secur.* **10**, 2669–2680. 10.1109/TIFS.2015.2473815 (2015).

[31] Holub, V., Fridrich, J. & Denemark, T. Universal distortion function for steganography in an arbitrary domain. *EURASIP J. Inf. Secur.* **1**, 1–13. 10.1186/1687-417X-2014-1 (2014).

